# Redirecting T cells to glypican-3 with 28.41BB.ζ and 28.ζ-41BBL CARs for hepatocellular carcinoma treatment

**DOI:** 10.1007/s13238-017-0489-0

**Published:** 2017-11-15

**Authors:** Haili Ma, Siye Chen, Yan He, Jingwei Huang, Yanhong Xu, Chao Wang, Cheng Lei, Ting Lu, Shengdong Xiao, Jinming Mao, Yiyun Xu, Hao Guo, Bohua Li, Minghui Zhang, Xiaowen He

**Affiliations:** 1 Department of Discovery, Origincell Co, Shanghai, 201203 China; 2 Institute of Immunology, School of Medicine, Tsinghua University, Beijing, 10084 China

**Dear Editor,**

T cells genetically engineered to express CARs specific for CD19 have shown breakthrough clinical successes in patients with B-cell lymphoid malignancies in recent years (Grupp SA et al., 2013; Kalos M et al., 2011; Maude SL et al., 2014). The technology holds great promise for other types of cancer, including solid tumors for which conventional cytoreductive therapies often fail (Kohler BA et al., 2015). However, the early clinical testing of CAR T cells against solid tumors has thus far benefited only a small fraction of patients (Ahmed N et al., 2015; Pule MA et al., 2008; Gilham DE et al., 2012), highlighting the need to explore novel antigens and to optimize antigen-specific CAR design.

Glypican-3 (GPC3), a membrane-bound proteoglycan, is expressed in several solid tumors including hepatocellular carcinoma (HCC) (Gao H et al., 2014). GPC3 is an attractive target for immunotherapy since it is not expressed at detectable levels in non-malignant tissues including normal or cirrhotic liver (Gao H et al., 2014). Recent early phase clinical trials tested a GPC3-specific GC33 mAb and demonstrated that targeting GPC3 is safe, well tolerated and, depending on the density of GPC3 expression on tumor cells, can achieve significant antitumor responses in patients with advanced HCC (Zhu AX et al., 2013).

Unlike clinically tested CD19-CARs, which contained either CD28 or 4-1BB co-stimulatory endodomains, the GPC3-CAR construct reported in these studies contained both CD28 and 4-1BB endodomains (Gao H et al., 2014). Li et al. evaluated a series of CAR constructs targeting GPC3, and GBBz was picked out as the best choice for its Th2 cytokine polarization profile (Li W et al., 2016). Recently, Zhao et al. compared several constructs targeting CD19. By using an *in vivo* ‘‘stress test’’, 1928z-41BBL provided the highest therapeutic efficacy, showing balanced tumoricidal function, increased T cell persistence, and decreased exhaustion (Zhao et al., 2015). In this study, for the first time we evaluated the anti-tumor properties of G3-28BBz or G3-28z-41BBL-modified T cells in a solid tumor—HCC.

To perform the study the synthesized G3-28BBz or G3-28z-41BBL CAR gene was cloned into lentivirus vector. G3-28BBz CAR encoded CD3ζ with costimulatory domains derived from CD28 and 4-1BB, and G3-28z-41BBL CAR encoded CD3ζ with costimulatory domains derived from CD28 and a separate 4-1BB ligand spaced by internal ribosome entry site (IRES) sequence (Fig. 1A). Immobilized CD3 antibody-activated T cells were transduced with prepared lentivirus vector encoding the indicated CAR construct. Cell surface expression of CARs was measured by flow cytometry (Fig. 1B and 1C). Both CARs were efficiently expressed on the cell surface of T cells without significant differences between the two constructs (Fig. 1C). The generated CAR-T cells contained >95% CD3-positive T cells, which comprises CD4- and CD8-positive T-cell subsets, and has the similar CD4/CD8 ratio as in non-transduced T cells (Fig. 1D).

**Figure 1:**
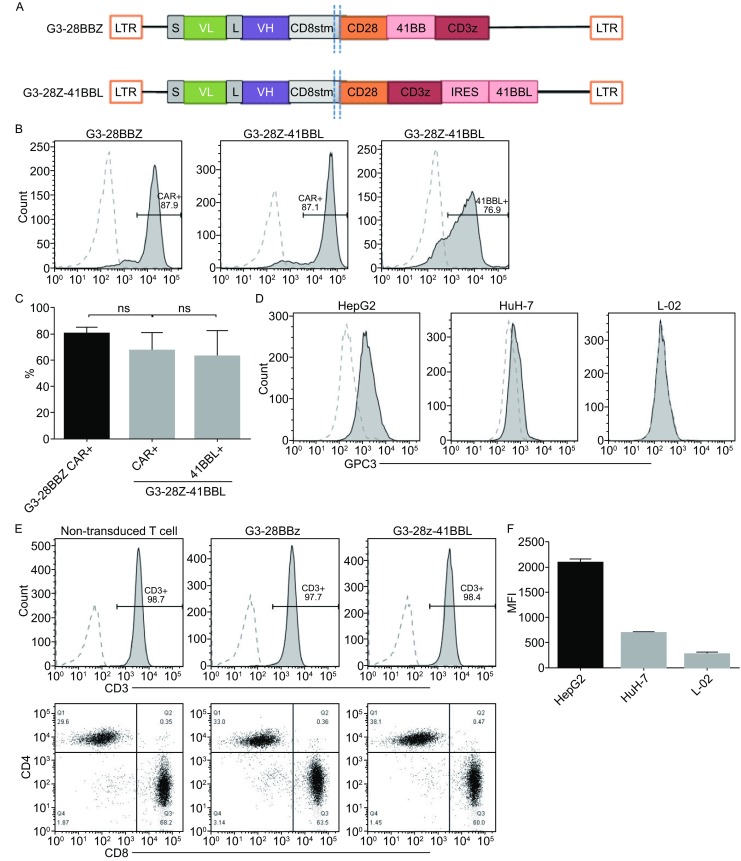

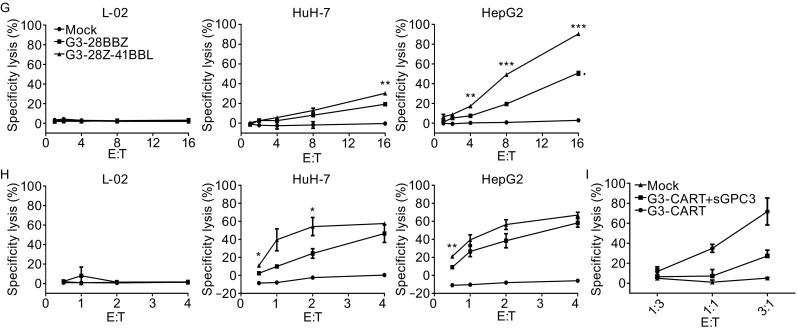
**Expression of GPC3-CAR on the surface of T cells and GPC3-CAR T cells recognize and kill GPC3-positive tumor cells**. (A) Schematic map of CAR constructs. LTR, long terminal repeat; S, signal sequence of CD8; VL-L-VH, single chain variable regions of monoclonal anti-GPC3 antibody; CD8 stem, part of the extracellular region and all of the transmembrane of CD8; CD28, intracellular signaling domain of CD28; 41BB, intracellular signaling domain of 4-1BB; CD3z, the entire cytoplasmic region of the TCR-ζ molecule; IRES, internal ribosome entry site; 41BBL, entire 4-1BB ligand molecule. (B and C) GPC3-CAR expression on T cell surface determined by flow cytometry. One representative donor and summary data for three independent donors. Mock T cells served as controls. No difference was detected between the expression levels of GPC3 CARs or 4-1BB ligand (ANOVA); (D) Generated CAR-T cells profiling for CD3/CD8/CD4 ratio by flow cytometry; (E and F) Flow cytometry measured GPC3 expression on HepG2, HuH-7, and L-02 cells; (G and H) Tumor cell lysis was measured with LDH release assay at indicated effector to target ratios and coculture for 4 h (G) or 18 h (H) against GPC3-positive solid tumor cell lines HepG2, HuH-7, and GPC3-negative immortal hepatic cell line L-02; (I) Adding soluble recombinant GPC3 protein at 5 μg/mL inhibited the killing activity. Representative results from 3 independent experiments were showed. One-way ANOVA with Tukey’s test was used for comparative analysis, *P < 0.05; **P < 0.01; ***P < 0.001

To test the cytotoxicity of G3-28BBz and G3-28z-41BBL CAR T cells towards GPC3-positive targets, two HCC cell lines (HepG2 and HuH-7) and one immortal hepatic cell line (L-02) have been tested for cell surface expression of GPC3 by flow cytometry. Except for L-02 cell line, both HepG2 and HuH-7 HCC cells expressed GPC3, but the expression level of GPC3 on HepG2 was more than that of HuH-7 (Fig. 1E and 1F). For GPC3 CAR-T cells cytotoxicity test, LDH release assays have been performed at the indicated effector to target ratios. Results showed that GPC3-CAR T cells specifically killed GPC3 positive HepG2 and HuH-7 cells, but not the GPC3 negative L-02 cells. When comparing the cytolytic activity between G3-28BBz and G3-28z-41BBL CAR T cells, the latter showed more potent cytolytic activity, especially towards GPC3 high positive HepG2 cells at hour 4 (Fig. 1G and 1H). In addition, mock T cells did not kill any of the target cells and the killing activity of CAR-T cells also can be inhibited by the addition of soluble recombinant human GPC3 protein, demonstrating that the cytolytic activity of T cells depends on the expression of CARs specifically targeting GPC3 (Fig. 1G–I).

In the *in vitro* cytokine release assay, the ability of GPC3 CAR-T cells to secrete cytokines has been evaluated. HepG2 and HuH-7 effectively induced cytokine production of both two GPC3 CAR-T cells, but not L-02 cells and the groups containing mock T cells. Striking differences were noted in cytokine release between G3-28BBz and G3-28z-41BBL CAR T cells, the latter produced higher level of cytokines except for IL-4. Moreover, in both of these two CAR-T cells, the cytokine production level of CCL3, CCL4, and IL-10 is much lower than that of IL-2, IL-6, IL-8, IFNγ and TNFα, especially for IL-10 (Fig. 2A). To further confirm the cytokine production differences between G3-28BBz and G3-28z-41BBL CAR-T cells, the serum cytokine levels of HuH-7 tumor-bearing NCG mice were compared. In the *in vivo* assays, the significant differences in the expression level of these cytokines were also observed between G3-28BBz and G3-28z-41BBL CAR-T cells, except for IL-10 (Fig. 2B). Moreover, the release of IFNα and IFNβ1 was also detected, although no difference was observed for IFNα production *in vitro* between G3-28BBz and G3-28z-41BBL CAR-T cells, IFNβ1 level increased significantly in G3-28z-41BBL CAR-T cells *in vitro* and *in vivo* as compared with G3-28BBz CAR-T cells (Fig. 2C and 2D). The potent antitumor activity of CD19-28z-41BBL also was attributable to be the enhanced IFNβ secretion (Zhao et al., 2015).

**Figure 2:**
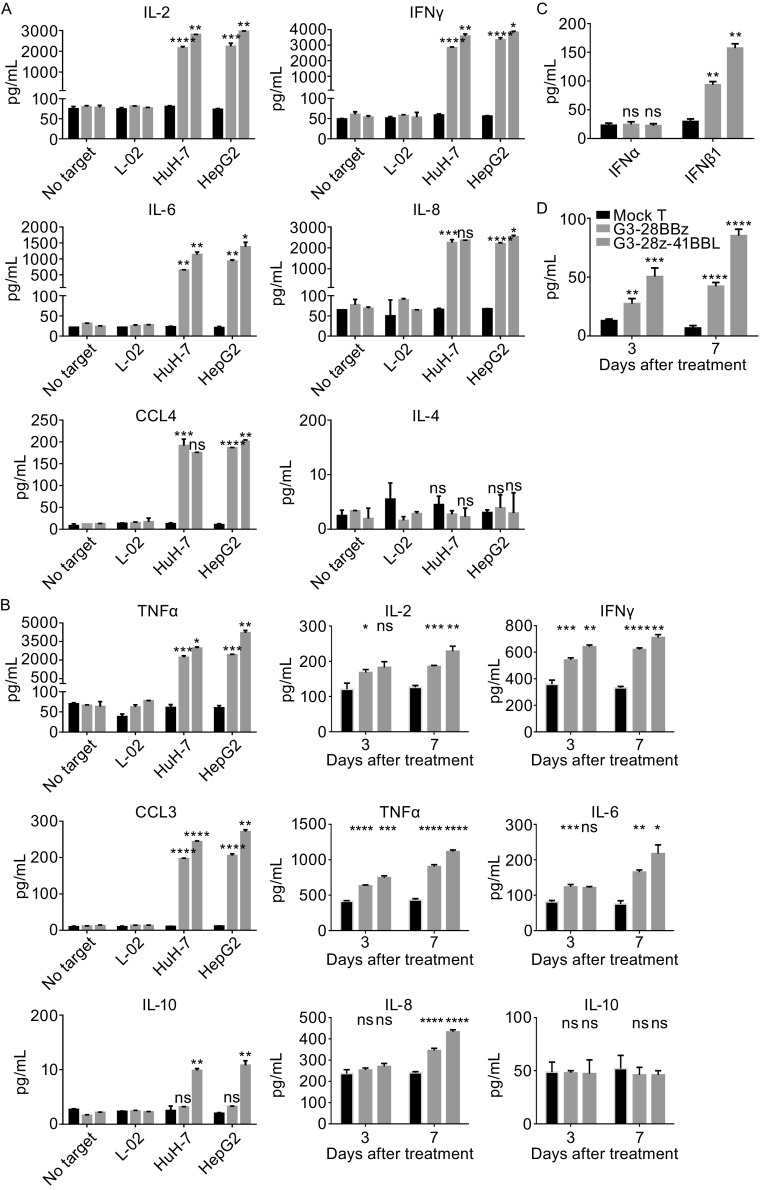

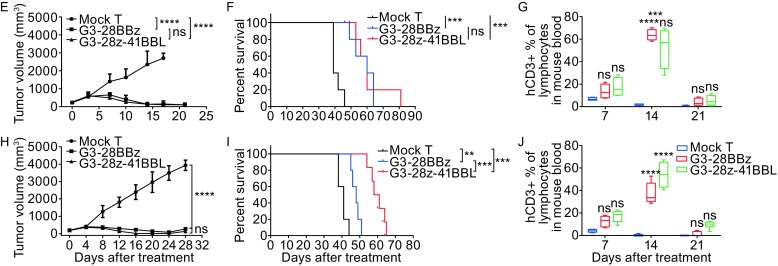
**Cytokines release and antitumor activity of GPC3 CAR-T cells** ***in vitro*** **and** ***in vivo***. (A) *In vitro* cytokine release by GPC3 CAR-T cells. GPC3-positive HepG2, HuH-7 and GPC3-negative L-02 cells were co-cultured with G3-28BBz/G3-28z-41BBL CAR or mock T cells for 24 h at 1:1 ratio and indicated cytokine levels in tissue culture supernatant were measured by Multiplex Luminex. (B) *In vivo* cytokine release by GPC3CAR-T cells. HuH-7 tumor-bearing NCG mouse was injected single dose of GPC3 CAR-T or mock T cells at 1 × 10^7^ CAR-T cells per mouse i.v., at Day 3 and Day 7, the serum were collected and pooled for cytokine levels measurement by Multiplex Luminex. (C) IFNα and IFNβ1 release was also test in the co-culture supernatant of HuH-7 and G3-28BBz/G3-28z-41BBL CAR or mock T cells, and (D) the serum level of IFNβ1. Mean and SD are shown. (E) Growth curve of HuH-7 xenografts treated with the indicated GPC3 CAR-T cells or mock T cells at high dose (1 × 10^7^ cells/mouse). The residual tumors treated with G3-28BBZ and G3-28Z-41BBL CAR-T cells were significantly smaller than those in mock T cells groups at Day 17. (F) Survival curve of HuH-7 tumor-bearing mice after treatment with high dose GPC3 CAR-T cells. (G) Measurement of human CD3^+^ T cells in mice PBMC, high dose. 20 μL of blood (treated with Heparin sodium) from each mouse was collected for flow cytometry test respectively at Day 7, 14 or 21 after treatment, the percentage ratios of human CD3^+^ T cells to mouse lymphocytes in peripheral blood were showed. (H) Growth curve of HuH-7 xenografts treated with the indicated GPC3 CAR-T cells or mock T cells at middle dose (3 × 10^6^ cells/mouse). (I) Survival curve of HuH-7 tumor-bearing mice after treatment with middle dose GPC3 CAR-T cells. (J) Measurement of human CD3^+^ T cells in mice PBMC, middle dose. One-way or two-way ANOVA with Tukey’s test was used for comparative analysis, Log-rank (Mantel-Cox) test for survival analysis, *P < 0.05; **P < 0.01; ***P < 0.001, ****P < 0.0001

To evaluate the *in vivo* therapeutic potential of GPC3 CAR-T cells, the highly immunodeficient mice (NCG) were injected subcutaneously with 1 × 10^7^ HuH-7 cells followed by injection of 1 × 10^7^ G3-28BBz, G3-28z-41BBL, or mock T cells on day 14 i.v. By measuring the tumor dimension every 3 or 4 days with calipers, GPC3 CAR-T cells significantly suppressed the growth of HuH-7 tumors and prolonged the mice survival in comparison to the mock group (Fig. 2E and 2F). The measurement of human CD3^+^ T cells in mice peripheral blood at 7, 14 or 21 days after the injection of T cells showed that the GPC3 CAR-T cells persisted for about three weeks and the amount of GPC3 CAR-T cells peaked at ~day 14 (Fig. 2G). Since we could not find significant differences between abilities of G3-28BBz and G3-28z-41BBL to suppress tumor growth and to prolong mice survival, a ‘‘stress test’’ was run by decreasing the dose of GPC3 CAR-T cells by 10-fold to 1 × 10^6^ cells/mouse. Although the tumor growth suppression is not observed because of the excessively decreased cell number, G3-28z-41BBL prolonged the mice survival compared with G3-28BBz and mock groups (Data not shown). For another optimized ‘‘stress test’’, a middle dose of GPC3 CAR-T cells (3 × 10^6^ cells/mouse) was injected, the growth of HuH-7 tumors were suppressed significantly as well as the high dose (Fig. 2H). Although G3-28BBz and G3-28z-41BBL CAR-T cells displayed a similar capability to inhibit the growth of tumor, the latter was significantly more effective in prolonging the survival of the tumor-bearing mice (Fig. 2I). Flow cytometry also showed that G3-28z-41BBL CAR-T cells had more robust proliferation (Fig. 2J).

The early phase testing of CAR-T cells in patients with B-cell leukemia and lymphomas have generated impressive results. More promisingly, Zhao et al. recently have compared a series of CD19-CARs with various 4-1BB and CD28 costimulatory signal combinations, one of which with separate entire molecule of 4-1BB ligand provided the highest therapeutic efficacy even at a really low dose (Zhao et al., 2015). Given the more modest efficacy in patients with solid tumors, we decided to evaluate this technology for HCC solid tumor. We generated two of GPC3-CARs and compared their antitumor potential against GPC3-positive solid tumors *in vitro* and *in vivo* with the goal of defining the CARs with the most promising antitumor properties for further clinical development. We found that GPC3-CARs containing the full length of 4-1BB ligand more than 4-1BB endodomain endowed T cells more potent cytolytic activity toward GPC3-positive HCC cells *in vitro*, and made T cells to produce higher level proinflammatory cytokines and chemokines, but not anti-proinflammatory cytokines *in vitro* and *in vivo*. Furthermore, both two GPC3-CARs exhibited robust antitumor activity in HCC xenografts in tumor-bearing mouse model at high dose. Although the tumor growth suppression was not observed when treated with a lower dose, the GPC3-CARs comprising the full length of 4-1BB ligand still prolonged the median survival of the tumor-bearing mice by ~20% as compared with GPC3-CARs containing 4-1BB endodomain (Data not shown). For another optimized ‘‘stress test’’, a middle dose of GPC3 CAR-T cells was injected, although G3-28BBz and G3-28z-41BBL CAR-T cells displayed a similar capability to inhibit the growth of tumor, the latter was significantly more effective in prolonging the survival of the tumor-bearing mice.

Most recently, Li et al. (2016) evaluated a set of CAR constructs targeting GPC3, both GBBz and G28BBz have the same potent antitumor activity, but given the latter produced higher amount Th2-biased IL-4 and IL-10, which may suppress anti-tumor immune responses (Sato T et al., 2011; Mannino MH et al., 2015), they chose GBBz for further clinical development (Li W et al., 2016). For the first time we evaluated the efficacy of GPC3-CAR with 4-1BB ligand for solid tumor treatment, we found that the separate entire 4-1BB ligand in GPC3-CAR induced higher amount Th1 cytokines (IL-2, IFNγ, TNFα, IL-6) than the endodomain counterpart in GPC3-CAR *in vitro* and *in vivo*. There is a wealth of preclinical studies and some clinical evidence to show that the production of these Th1-typecytokines correlates with anti-tumor responses (Dredge K et al., 2015). Higher level release of chemokines (IL-8, CCL3, CCL4) has also been noted by GPC3-CAR with full length of 4-1BB ligand, which may be beneficial to anti-tumor responses by recruiting T cell and monocyte accumulation at sites of inflammation (Taub DD et al., 1996). Additionally, we measured the production of Th2 cytokines IL-4 and IL-10 too, but no more release of IL-4 was observed from GPC3 CAR-T cells than mock T cells. In contrast, more IL-10 was released by G3-28Z-41BBL CAR-T cells following induction with GPC3 positive HCC cells. Although the difference *in vitro* is statistically significant, the amount of IL-10 is relatively low so that we didn’t confirm the difference *in vivo*.

In conclusion, G3-28z-41BBL CAR construct with separate entire 4-1BB ligand rendered T cells a more potent cytolytic activity, higher amount production ability of proinflammatory cytokines/chemokines, and superior therapeutic activity in inhibiting the GPC3-positive tumors and prolonging tumor-bearing mouse survival *in vivo*, suggesting that it may have the potential to be developed as a promising agent for HCC.

## **FOOTNOTES**

Haili Ma, Siye Chen, Yan He, Jingwei Huang, Yanhong Xu, Chao Wang, Cheng Lei, Ting Lu, Shengdong Xiao, Jinming Mao, Yiyun Xu, Hao Guo, Bohua Li, Minghui Zhang, and Xiaowen He declare that they have no conflict of interest. This article does not contain any studies with human subjects performed by the any of the authors. All institutional and national guidelines for the care and use of laboratory animals were followed.

## Electronic supplementary material

Below is the link to the electronic supplementary material.

Supplementary material 1 (PDF 190 kb)

## References

[CR1] Ahmed N, Brawley VS, Hegde M (2015). Human Epidermal Growth Factor Receptor 2 (HER2)-Specific Chimeric Antigen Receptor-Modified T Cells for the Immunotherapy of HER2-Positive Sarcoma. J Clin Oncol.

[CR2] Dredge K, Marriott JB, Todryk SM, Dalgleish AG (2015). Adjuvants and the promotion of Th1-type cytokines in tumour immunotherapy. Cancer Immunol Immunother.

[CR3] Gao H, Li K, Tu H (2014). Development of T cells redirected to glypican-3 for the treatment of hepatocellular carcinoma. Clin Cancer Res.

[CR4] Gilham DE, Debets R, Pule M, Hawkins RE, Abken H (2012). CAR–T cells and solid tumors: tuning T cells to challenge an inveterate foe. Trends Mol Med.

[CR5] Grupp SA, Kalos M, Barrett D (2013). Chimeric antigen receptor-modified T cells for acute lymphoid leukemia. N Engl J Med.

[CR6] Kalos M, Levine BL, Porter DL (2011). T cells with chimeric antigen receptors have potent antitumor effects and can establish memory in patients with advanced leukemia. Sci Transl Med..

[CR7] Kohler BA, Sherman RL, Howlader N, et al. (2015) Annual Report to the Nation on the Status of Cancer, 1975-2011, featuring incidence of breast cancer subtypes by race/ethnicity, poverty, and state. J Natl Cancer Inst107(6) 10.1093/jnci/djv048 PMC4603551 25825511

[CR8] Li W, Guo L, Rathi P, Marinova E, Gao X, Wu MF, Liu H, Dotti G, Gottschalk S,Metelitsa LS,Heczey A (2016) Redirecting T Cells toGlypican-3 with 4-1BB Zeta Chimeric Antigen Receptors Results in Th1 Polarization and Potent Antitumor Activity. Hum Gene Ther 2016 Aug 16 10.1089/hum.2016.025 PMC5444493 27530312

[CR9] Mannino MH, Zhu Z, Xiao H, Bai Q, Wakefield MR, Fang Y (2015). The paradoxical role of IL-10 in immunity and cancer. Cancer Lett.

[CR10] Maude SL, Frey N, Shaw PA (2014). Chimeric antigen receptor T cells for sustained remissions in leukemia. N Engl J Med.

[CR11] Pule MA, Savoldo B, Myers GD (2008). Virus-specific T cells engineered to coexpress tumor-specific receptors: persistence andantitumor activity in individuals with neuroblastoma. Nat Med.

[CR12] Sato T, Terai M, Tamura Y, Alexeev V, Mastrangelo M, Selvan S (2011). Interleukin 10 in the tumor microenvironment: a target for anticancer immunotherapy. Immunol Res.

[CR13] Taub DD, Anver M, Oppenheim JJ, Longo DL, Murphy WJ (1996). T lymphocyte recruitment by interleukin-8 (IL-8). IL-8-induced degranulation of neutrophils releases potent chemoattractants for human T lymphocytes both in vitro and in vivo. J Clin Invest..

[CR14] Zhao Zeguo, Condomines Maud, van der Stegen Sjoukje JC, Perna Fabiana, Kloss Christopher C, Gunset Gertrude, Plotkin Jason, Sadelain Michel (2015). Structural Design of Engineered Costimulation Determines Tumor Rejection Kinetics and Persistence of CAR T Cells. Cancer Cell.

[CR15] Zhu AX, Gold PJ, El-Khoueiry AB (2013). First-in-man phase I study of GC33, a novel recombinant humanized antibody against glypican-3, in patients with advanced hepatocellular carcinoma. Clin Cancer Res.

