# Supplementary material for: Redirecting T cells to glypican-3 with 28.41BB.ζ and 28.ζ-41BBL CARs for hepatocellular carcinoma treatment
Source: Protein Cell. 2017 Nov 15;9(7):664–9. doi: 10.1007/s13238-017-0489-0 (PMC6019662; doi:10.1007/s13238-017-0489-0)
Supplement: Supplementary file 1 — Supplementary material 1 (PDF 190 kb) [file 13238_2017_489_MOESM1_ESM.pdf]

SUPPLEMENTAL MATERIALS

MATERIALS AND METHODS

Cell lines

HepG2, HuH-7 andL-02 cell lines were purchased from the Type Culture Collection of Chinese Academy of Sciences (Chinese Academy of Sciences, Shanghai). These cell lines were tested and authenticated by DNA profiling for polymorphic short tandem repeat markers. All cells were maintained according to the manufacturer's manual and were routinely tested for mycoplasma contamination.

Generation of lentiviral constructs

Genes were synthesized by GenScript (Nanjing, CN) encoding the GPC3-specific scFv, CD8a signal peptide, short hinge and transmembrane domain, also CD28.4-1BB.CD3ζ signaling domains for G28BBz, CD28.CD3ζ and a separate 4-1BB ligand spaced by IRES forG28z-41BBL (**Fig 1A**). The GPC3-specific scFv is an optimized sequence which has been selected from our phage library platform. Both genes contained a NotI site at the 5' end and a ClaI site at the 3' end and were subcloned into the 2ndgeneration self-inactivating EF-1a promoter-based lentiviral expression vector pHage-EF1a-MCS (constructed in-house by our lab).The sequence of each cloned CAR was verified by sequencing (Sanny, Shanghai).

Lentivirus production

Recombinant lentiviral particles were produced by co-transfection of 293FT package cell line by using a Lipofectamine 2000 transfection system (Invitrogen, Cat #: 11668027).

Generation of CAR-T cells

Human peripheral blood mononuclear cells (PBMCs) isolated from healthy volunteer donors were stimulated inhuman CD3antibody-coated plates, the human CD3 antibody (RnD Systems, Cat #: MAB100-500) was diluted in PBS to a concentration of 10μg/m Land was used to coat plates for 2 hours at 37℃ or overnight at 4℃. The PBMCs were activated for 48hrs in complete RPMI (Gibco RPMI 1640, 10% heat inactivated fetal bovine serum and 2mM Glutamax) with IL-2 (1000IU/mL) (RnD Systems, Cat #: 202-IL-500). T cells were then transduced with the lentiviral vector at a multiplicity of infection of 8 U/cell. The transduced T cells were cultured at a concentration of 1x 10^6 cells/mL in the presence of recombinant human IL-2 (300 U/mL) every other day.

Flow cytometry

For all flow cytometry analyses, BD FACSCanto™ II was used (BD Biosciences). Results were analyzed by FlowJo (FlowJo) or BD FACSDiva™ Software (BD Biosciences). GPC3-CAR expression was detected by recombinant human GPC3-His (Sino Biological Inc., Cat #: 10088-H08H-50) and Anti-6X His tag® antibody DyLight® 650 (abcam, Cat #: ab117504). 4-1BB ligand expression on the surface of

T cell was detected by recombinant human 4-1BB-His (Sino Biological Inc., Cat #: 10041-H08H-50) and Anti-6X His tag® antibody (DyLight® 650). GPC3 expression of tumor cell lines was detected with Glypican 3 Monoclonal Antibody (SP86) (Invitrogen, Cat#: MA5-16368) and Goat anti-Rabbit IgG (H+L) Highly Cross-Adsorbed Secondary Antibody, Alexa Fluor 488 (Invitrogen, Cat #: A-11034), Rabbit IgG Isotype Control (Invitrogen,Cat#: 10500C) served as negative control. FITC Mouse Anti-Human CD3 (BD Pharmingen, Cat#:555339), APC-H7 Mouse Anti-Human CD4 (BD Pharmingen, Cat#:560158) and PE anti-human CD8 Antibody (BioLegend, Cat#:344706) were used for human CD3/CD4/CD8 detection on T cells. APC Mouse Anti-Human CD3 BD (BD Pharmingen, Cat#:340440) and PerCP anti-human CD45 Antibody (BioLegend, Cat#:304026) were used for human T cells measurement in mouse peripheral blood. FITC Mouse IgG2a, κ Isotype Control (BD Pharmingen, Cat#:553456), APC-H7 Mouse IgG1, κ Isotype Control (BD Pharmingen, Cat#:560167), PE Mouse IgG1, κ Isotype Control (BD Pharmingen, Cat#:550617), APC Mouse IgG1 κ Isotype Control (BD Pharmingen, Cat#:554681) and PerCP Mouse IgG1 κ Isotype Control (BD Pharmingen, Cat#:559425) served as negative control.

Cytotoxicity assays in vitro

The specific cytotoxicity of CAR-modified T cells toward various HCC cells at the different effector: target ratios of 16:1, 8:1, 4:1, 2:1and 1:1 was measured after coculturing for 4 hours or ratios of 4:1, 2:1, 1:1 and 0.5:1 for 18hours by lactate

dehydrogenase assay using the CytoTox96 nonradioactive cytotoxicity kit (Promega, Cat#:G1780) according to the manufacturer's instructions.

Multiplex Cytokine quantification

GPC3-CAR T cells were co-cultured at 1:1 ratio with target cells and supernatant was collected at 24hrs. Samples were analyzed with Cytokine/Chemokine Human for Luminex™ Platform (Invitrogen, Cat#:LHC4031M, LHC0101M, LHC0021M, LHC0041M, LHC0061M, LHC0081M, LHC1021M, LHC1051M, LHC3011M, EPX01A-10216-901, EPX01A-12088-901) according to the manufacturer's manual.

Xenograft models of human hepatocellularcarcinoma

5 to 6-week-old NOD-Prkdc^{em26Cd52}Il2rg^{em26Cd22}/Nju (NCG) female mice (NBRI, Nanjing, CN) were housed and treated under specific pathogen-free conditions at the Experimental Animal Center of Shanghai Fudan University School of Medicine (Shanghai, China). All animal experiments were carried out according to the protocols approved by the Shanghai Medical Experimental Animal Care Commission. For the established subcutaneous (s.c.) Huh-7 models, mice were inoculated subcutaneously with 1x 10^7 Huh-7 cells on the right flank on day 0. When the tumor burden was approximately 200 to300 mm^3, mice were randomly allocated into three groups (n=5 or 6) and assigned to receive one of the following injections: (i) genetically modified aGPC3-28BBZ CART cells dissolved in sterile saline (G28BBZ); (ii) genetically modified aGPC3-28Z-41BBL CAR T cells in sterile saline (G28Z-41BBL); and (iii)

genetically modified mock T cells in sterile saline (Mock). CAR T cells were intravenously (i.v.) injected according to indicated doses. Tumor dimensions were measured with calipers, and tumor volumes were calculated using the formula V = 1/2 (length x width^2).

Statistical analysis

All experiments were repeated three times except the in vivo animal assay, and representative results have been shown. Statistical analyses were performed using Prism GraphPad software. For studies comparing two groups we used an unpaired Student's *t*-test. For studies with multiple groups, we used a one-way or two-way ANOVA followed by Tukey's test which accounts for multiple comparisons. All analyses were two-tailed. The mouse survive curves was analyzed using the Kaplan-Meier method and compared using the Log-rank (Mantel-Cox) test.
